# Adherence and Persistence with First-Line Therapy and Compliance with Glaucoma Guidelines Using Japanese Health Care/Pharmacy Claims Database

**DOI:** 10.1089/jop.2020.0096

**Published:** 2021-01-11

**Authors:** Chikako Shirai, Nobushige Matsuoka, Toru Nakazawa

**Affiliations:** ^1^ Medical Affairs, Pfizer Japan, Inc., Tokyo, Japan.; ^2^ Biometrics & Data Management, Pfizer R&D Japan G.K., Tokyo, Japan.; ^3^ Department of Ophthalmology, Tohoku University Graduate School of Medicine, Sendai, Japan.

**Keywords:** adherence, persistence, glaucoma, fixed-combination therapy, claims data, latanoprost

## Abstract

***Purpose:*** This study evaluated Japanese patients' adherence to first-line therapy and physicians' compliance with the guidelines (GLs) for glaucoma in daily practice.

***Methods:*** This retrospective, noninterventional cohort study used a Japanese health care/pharmacy claims database from 2011 to 2016. We compared adherence based on the primary outcomes assessed as proportion of days covered (PDC) and persistence between patients who received first-line monotherapy followed by second-line fixed-combination therapy (GL-compliant cohort) and those who received first-line fixed-combination therapy (GL-noncompliant cohort). Furthermore, we explored treatment patterns, glaucoma consultation, and key factors associated with GL noncompliance.

***Results:*** Among 11,666 patients who received first-line therapy, 1,183 (10.1%) and 542 (4.6%) were in the GL-compliant and GL-noncompliant cohorts, respectively. Prostaglandin (70.7%) and subsequent prostaglandin/β blocker (BB) (20.9%) within 12 months were most used by the GL-compliant cohort. Prostaglandin/BB (48.9%) and carbonic anhydrase inhibitor/BB (51.1%) were prescribed for the GL-noncompliant cohort. The mean PDC [standard deviation (SD)], persistence rate (95% confidence interval), and consultation (SD) over 12 months were 60.9% (34.0), 16.0% (14.0–18.1), and 5.23 (3.21) times, respectively, in the GL-compliant cohort and 59.7% (35.8), 22.0% (18.6–25.5), and 4.76 (3.19) times, respectively, in the GL-noncompliant cohort. No significant differences were observed between the 2 cohorts. No clinically relevant factor associated with GL noncompliance was found.

***Conclusions:*** Around 5% of patients were prescribed a fixed-combination eye drop as first-line therapy not in accordance with GLs. The similarity of adherence and persistence between the 2 cohorts indicates that first-line fixed-combination therapy could be considered for glaucoma treatment.

## Introduction

Glaucoma is the most common causative disease of blindness in Japan. The proportion of patients with blindness caused by glaucoma increased to 28.6% in the fiscal year 2015 from previous surveys in the fiscal years 2007–2009.^1^ Visual field deterioration can be prevented if glaucoma is detected at an early stage, and therapy is initiated soon after diagnosis.^2^ However, the Tajimi Study, conducted by the Japan Glaucoma Society, showed that ∼90% of glaucoma cases were undiagnosed despite a prevalence of 5.0% in Japanese people aged >40 years,^3^ indicating that therapy was not started in a timely manner.

The Japan Glaucoma Society Guidelines for Glaucoma (4th edition) state that reduction of intraocular pressure (IOP) is the only established treatment for primary open-angle glaucoma (broad definition of glaucoma), and the guidelines (GLs) recommend starting with a single drug (monotherapy) as first-line therapy, 2 drugs as second-line therapy, increasing by one drug each time, followed by laser treatment or surgery.^4^ However, some patients may be better to start receiving multidrug or laser treatment since it is also known that the disease may continue to progress during the late stages of glaucoma, even when treatment is provided. Accordingly, early detection and treatment of glaucoma are crucial. A recent study reported that selective laser trabeculoplasty was more cost-effective than eye drop treatment as a first-line therapy,^5^ although the medical costs vary from country to country.

Adherence to eye drops is clinically critical and is influenced by the number of eye drops used in daily practice. A previous 6-month observational trial reported that a fixed-combination (latanoprost/timolol) therapy led to a superior adherence rate and ocular surface health compared with unfixed-combination (latanoprost and timolol) therapy.^6^ Another randomized clinical trial demonstrated that fixed-combination therapy (travoprost/timolol) improved long-term adherence of patients with glaucoma compared with unfixed therapy (travoprost and timolol).^7^ Our database study also demonstrated that fixed-combination therapy was associated with greater adherence than unfixed therapy.^8^

Specifically, latanoprost/timolol fixed-combination therapy for glaucoma has a high adherence rate. It lowers IOP more potently than unfixed-combination latanoprost or timolol monotherapy,^9^ and was developed with the aim of reducing the number of eye drops (reducing patient burden), avoiding the washout effect due to an inadequate interval of instillation time (reducing the drug's efficacy), and reducing preservative exposure (reducing the risk of ocular surface disease). As such, fixed-combination eye drops have advantages in glaucoma treatment and can be used as a first-line therapy at the physician's discretion in daily practice. However, since the release of the GLs, there have been no large-scale studies since in Japan on the state of GL compliance in clinical practice or adherence to first-line treatment.

Our primary objective was to compare patient adherence and persistence with first-line therapy between GL-compliant and GL-noncompliant cohorts using a Japanese administrative health care/pharmacy claims database. Secondary objectives were to explore physicians' compliance with the GLs for glaucoma, the pattern of annual glaucoma treatment, incidence of annual glaucoma consultation, incidence of annual glaucoma surgery, and key factors associated with GL noncompliance.

## Methods

### Study overview and ethics

This retrospective, observational cohort study used a commercially available Japanese health care/pharmacy claims database to investigate the prescription status in daily practice. The database did not include data for glaucoma severity (eg, IOP, visual field, pathology) that may influence the treatment course of glaucoma (monotherapy or fixed-combination therapy as a first-line therapy). To address this limitation, we designed the distribution of glaucoma severity to be as comparable as possible between the cohorts. Therefore, data for patients with glaucoma who were newly prescribed a topical IOP lowering medication as a first-line therapy of either monotherapy followed by second-line fixed-combination therapy (GL-compliant cohort) or first-line fixed-combination therapy (GL-noncompliant cohort) were extracted and compared between the cohorts.

Since this study used only anonymized data from patients and did not use information linkable to individual identification, ethical approval and informed consent from the patients were not required according to the “Ethical Guidelines for Medical and Health Research Involving Human Subjects”^10^ set out by the Japanese government. This research project adhered to the tenets of the Declaration of Helsinki.

### Data source and study cohort

The database was provided by MinaCare Co., Ltd., and secondary use of the data was permitted by each health insurance group. The MinaCare database contains anonymized data on both health checkup and medical/pharmacy claims of workers and their family members among a wide range of age groups <75 years.^11,12^ The database is regularly updated and has accumulated data since 2008 and, as of April 2017, included data from 6.3 million individuals, accounting for 1.7% of the Japanese population.^13^ Furthermore, it is generally consistent with 2 other national databases, and is useful as it has a low selection bias and a large sample size with a wide age distribution; however, it only targets large nationwide corporations and does not include individuals in primary industries or the self-employed.^11^

Patients with International Classification of Diseases, 10th Edition (ICD-10) diagnosis codes H401 (normal tension glaucoma, primary open-angle glaucoma, and open-angle glaucoma) and H409 (unspecified glaucoma) were extracted from the database between April 1, 2011 and March 31, 2016. Patient identification (anonymized), age, sex, body weight, height, body mass index (BMI), smoking status, ICD-10 diagnosis codes, name/date of any prescribed drugs, hospital location, name of glaucoma medication class, name/date of glaucoma diagnosis tests, name/date of glaucoma-related surgery, and health checkup data, such as anthropometric measurements, blood pressure, blood glucose level, blood lipid level, liver function test values, hematologic values, and urine test results from the health checkup database, were also extracted. The index date was defined as the date of first prescription of topical IOP lowering medication for each patient, and the study was divided into 2 periods, before and after the index date, to determine newly prescribed medication for each patient. Among the extracted patients with glaucoma, eligible patients included those who did not receive glaucoma treatment in the preindex period for ∼12 months, and thereafter had at least one record of prescription of topical glaucoma medication on or after the index date for ≥1 year. Topical glaucoma treatments included prostaglandin (PG) analog, β blocker (BB), carbonic anhydrase inhibitor (CAI), α2 receptor agonist (AA), α1 receptor blocker (AB), αβ blocker (ABB), cholinergic agonist, muscarinic agonist (MA), rho-associated coiled kinase inhibitor (ROCKI), fixed combinations and their generics. Patients with a history of glaucoma surgery, including laser treatment, on or before the index date were excluded.

The GL-compliant cohort was defined as patients who were newly prescribed a monotherapy as first-line therapy followed by a fixed-combination therapy as second-line therapy during the postindex period. Patients who switched to an unfixed-combination therapy were excluded. Patients who switched to other drug class monotherapies were considered adherent to first-line monotherapy. Patients who switched to a combination therapy were considered to have discontinued their treatment to start second-line therapy, indicating that they required more potent or appropriate medication. In contrast, the GL-noncompliant cohort included patients with a fixed-combination therapy as first-line therapy. Patients who switched to an unfixed-combination therapy or monotherapy were considered to have discontinued their treatment.

### Study outcomes

The primary outcomes of this study were adherence and persistence rates with the first-line topical glaucoma medication over the 12-month postindex period. Adherence was measured by the proportion of days covered (PDC) with medication. The *a priori* definition of the adherence cutoff point was 80%. Secondary outcomes were physicians' compliance with the GLs for glaucoma, the pattern of annual glaucoma treatment, incidence of annual glaucoma consultation, and incidence of annual glaucoma surgery. The key factors associated with noncompliance to local GLs were also explored using patients' demographic and clinical characteristics at the index date.

### Assessments

Patient background characteristics included sex, age, body weight, height, BMI, smoking status/history, health checkup results, comorbidity, and residency region. Residency regions were classified into 8 regions from Hokkaido to Kyushu, including Okinawa,^14^ 2 areas (East Japan vs. West Japan),^15^ and city size (big cities including Tokyo and 20 ordinance-designated cities compared with the others).^16^

PDC was calculated for first-line topical glaucoma medication (monotherapy or fixed-combination therapy), regardless of switching to other drug classes. To address possible variations in dosage and the prescribed period reported on a claim for glaucoma medication, adjustments were made based on the unit volume of the formulation. For example, if the dosage was “7.5” and the period was “1” in a claim, this was converted to “2.5 mL (approved bottle) × 3 bottles” prescribed for 3 months, since one bottle (2.5 mL for once-daily formulations or 5 mL twice-or-more-daily formulations) is sufficient for application to one eye for >30 days but corresponds to 30 days dispensed. In Japan, the recommended use by period of an opened bottle of eye drop is generally 4 weeks. Accordingly, patients were permitted a 30-day grace period to obtain the next prescription. In addition, if one prescription consisted of ≥7 bottles, the patient was considered as receiving treatment for both eyes. The PDC value was calculated as follows:


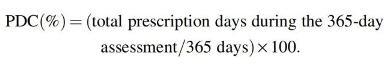


Persistence refers to the act of continuing index therapy (ie, first-line monotherapy or fixed-combination therapy). The definition of “persistence” is the duration in days from the index date (the first prescription date of the index therapy) to the last prescription date plus the number of prescription days or to the discontinuation date allowing a 30-day grace period. Treatment was considered as “discontinued” if there was no prescription record for >30 days or if the patient switched to second-line prescriptions. The cumulative discontinuation rate was analyzed based on the time to discontinuation and was estimated using the Kaplan–Meier method. Patients who continued the first-line treatment for 12 months were treated as censored at 12 months. The persistence rate at the evaluated time point was calculated as follows:

$$\begin{array}{cc} & Persistencerate\left(\%\right)=\\ & 100\%-cumulativediscontinuationrate. \end{array}$$

The annual consultation rate was calculated as the number of eye examinations per patient for 12 months after the index date. The examinations were categorized into 3 groups: (1) general eye examination including ophthalmoscopy (retina, optic disk, and vitreous humor tests) and tonometry (IOP test); (2) perimetry (visual field test); and (3) optical coherence tomography.

### Statistical analysis

Results were presented as mean and standard deviation (SD) or number and proportion of patients. The mean PDC for 12 months between 2 cohorts was compared using *t* test. In addition, the number and proportion (percentages) of patients in the following categories were identified by cohort: PDC ≥0.8; PDC <0.8; 0.6 ≤ PDC <0.8; 0.4 ≤ PDC <0.6; 0.2 ≤ PDC <0.4; and PDC <0.2. The proportion of patients with or without PDC ≥0.8 between the 2 cohorts was compared using Pearson's *χ*^2^ test.

Time (in days) to treatment switching or discontinuation over 12 months was analyzed using the Kaplan–Meier method, and the persistence rate at 6 and 12 months and the corresponding Wald 95% confidence intervals (CIs) were calculated. In addition, differences in persistence were compared using the log-rank test. Patients who did not switch to other medications or did not discontinue first-line medication were censored on the last day of the 12th month in the postindex period.

Bonferroni correction was used to adjust for multiplicity (*m* = 6, where *m* is the total number of statistical tests in this study). All *P* values >0.05/6 ( = 0.00833) were considered statistically significant for comparisons between cohorts.

Univariate logistic regression model was performed with subsequent multivariate analyses for those with *P* values <0.1, followed by variable selection using a stepwise manner to identify factors associated with GL noncompliance. All statistical analyses were performed using SAS version 9.4 (SAS Institute Inc., Cary, NC, USA).

## Results

**FIG. 1.**
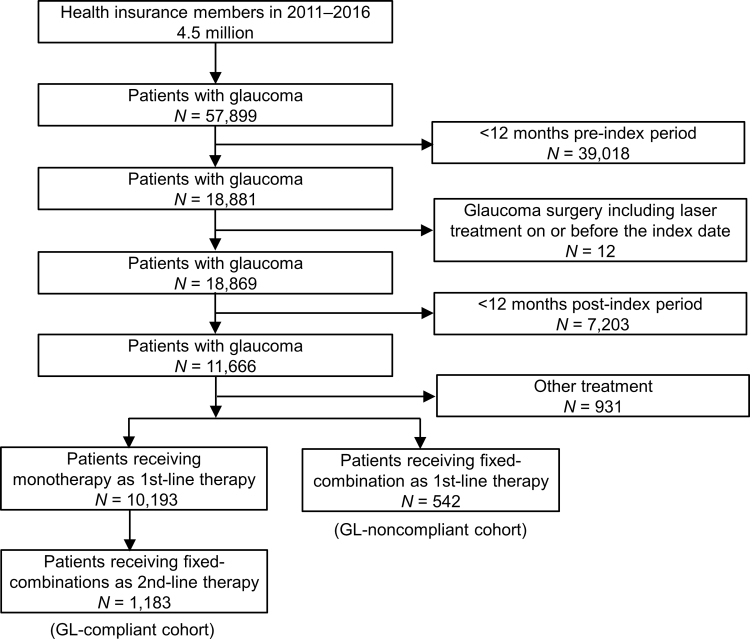
Flowchart of the data extraction.

A total of 57,899 patients were diagnosed with glaucoma in the database (Fig. 1). After excluding 39,030 patients with <12 months preindex period or glaucoma surgery on or before the index date, we extracted 18,869 patients prescribed with an IOP lowering eye drop as first-line therapy and analyzed 11,666 patients who had at least one record of prescription of topical glaucoma medication on or after the index date for ≥1 year. Among these patients, 10,193 (87.4%) received a monotherapy as first-line therapy and 1,183 (10.1%) subsequently switched to fixed-combination eye drops as a second-line therapy (GL-compliant cohort). Meanwhile, 542 (4.6%) patients received fixed-combination eye drops as first-line therapy (GL-noncompliant cohort). In addition, 931 (8.0%) received other treatments such as unfixed-combination eye drops as first-line therapy.

The baseline demographics and clinical characteristics of the 2 cohorts were similar (Table 1). The mean age of the patients was 52 years, and most patients had a moderate BMI, nonsmoking habit, and normal values for glucose and lipid metabolism and liver function. In addition, 13%–14% of patients received medication for other indications. More than 20% of each cohort had comorbidities, including hypertension, hyperlipidemia, diabetes mellitus, and cancer. In both cohorts, >70% of patients lived in East Japan and >80% of patients lived in large cities.

**Table 1.** Baseline Demographic and Clinical Characteristics of Subjects

Variable	GL-compliant cohort (*N = *1183)		GL-noncompliant cohort (*N = *542)	
	N	Percentage or mean ± SD	n	Percentage or mean ± SD
Sex, male (%)	666	56.3	322	59.4
Age (years)	1183	52.6 ± 11.2	542	52.3 ± 12.4
Body weight (kg)	659	63.7 ± 12.8	295	64.3 ± 13.2
Height (cm)	674	165.5 ± 8.8	302	165.6 ± 8.9
Body mass index (kg/m^2^)	659	23.1 ± 3.6	295	23.3 ± 3.8
Smoking (%)				
Yes	120	10.1	52	9.6
No	541	45.7	246	45.4
Medication for other indications (%)				
Yes	160	13.5	71	13.1
No	486	41.1	215	39.7
Fasting blood glucose (mg/dL)	632	99.0 ± 21.0	284	97.7 ± 17.1
HbA1c (NGSP)	612	5.7 ± 0.8	277	5.6 ± 0.8
Urine glucose (%)				
Negative (−)	629	53.2	277	51.1
Positive (≥±)	24	2.0	10	1.8
Urine protein (%)				
Negative (−)	600	50.7	277	51.1
Positive (≥±)	67	5.7	22	4.1
Antihypertensive drug (%)				
Yes	114	9.6	56	10.3
No	532	45.0	231	42.6
Systolic blood pressure (mm Hg)	674	122.3 ± 16.2	302	122.4 ± 16.4
Diastolic blood pressure (mm Hg)	674	76.4 ± 11.9	302	76.7 ± 11.8
Total cholesterol (mg/dL)	222	208.7 ± 34.5	92	205.8 ± 36.1
Triglycerides (mg/dL)	670	114.8 ± 82.7	301	112.6 ± 75.0
HDL-C (mg/dL)	671	62.9 ± 16.8	302	63.6 ± 16.9
LDL-C (mg/dL)	671	123.4 ± 29.0	302	121.1 ± 29.1
Aspartate aminotransferase (U/L)	672	23.3 ± 10.3	301	23.5 ± 12.3
Alanine aminotransferase (U/L)	672	23.8 ± 17.2	301	25.4 ± 20.2
*γ*-GTP (U/L)	671	39.4 ± 38.7	301	47.9 ± 70.3
Common comorbidities (%)				
Hypertension	294	24.9	139	25.6
Coronary artery disease	101	8.5	38	7.0
Angina pectoris	78	6.6	30	5.5
Acute myocardial infarction	20	1.7	5	1.0
Ischemic heart disease	8	0.7	8	1.5
Cardiac arrhythmia	54	4.6	27	5.0
Heart failure	49	4.1	26	4.8
Atherosclerosis or PAOD	103	8.7	51	9.4
Cerebral ischemia/chronic apoplexy	70	5.9	31	5.7
Cerebral infarction	43	3.6	23	4.2
Varicose veins of lower limb	4	0.3	3	0.6
Hyperlipidemia	310	26.2	147	27.1
Diabetes mellitus	296	25.0	143	26.4
Hypothyroidism	90	7.6	38	7.0
Water-electrolyte disorder	66	5.6	38	7.0
Mental disease or neurosis	105	8.9	42	7.7
Depression	48	4.1	23	4.2
Insomnia	115	9.7	59	10.9
Parkinson's disease	5	0.4	6	1.1
Dementia	1	0.1	3	0.6
Gastrointestinal ulcer	135	11.4	62	11.4
Nephropathy or chronic renopathy	42	3.6	25	4.6
Kidney failure	12	1.0	9	1.7
Incontinentia	1	0.1	1	0.2
Hepatopathy	142	12.0	69	12.7
Liver failure	1	0.1	1	0.2
Pneumonia	42	3.6	12	2.2
Asthma or COPD	164	13.9	92	17.0
Cancer	337	28.5	149	27.5
Anemia	106	9.0	37	6.8
Locations				
8 regions (%)				
Hokkaido	33	2.8	11	2.0
Tohoku	36	3.0	20	3.7
Kanto	771	65.2	362	66.8
Chubu	84	7.1	39	7.2
Kinki	123	10.4	60	11.1
Chugoku	33	2.8	14	2.6
Shikoku	11	0.9	6	1.1
Kyushu	92	7.8	30	5.5
Areas (%)				
East Japan	859	72.6	396	73.1
West Japan	324	27.4	146	26.9
City size (%)				
Tokyo and all GODMCs	970	82.0	455	83.9
Other than GODMCs	213	18.0	87	16.1

GL, guidelines; NGSP, National Glycohemoglobin Standardization Program; HDL-C, high-density lipoprotein cholesterol; LDL-C, low-density lipoprotein cholesterol; γ-GTP, γ-glutamyl transpeptidase; PAOD, peripheral arterial occlusive disease; COPD, chronic obstructive pulmonary disease; GODMCs, government ordinance-designed major cities.

**FIG. 2.**
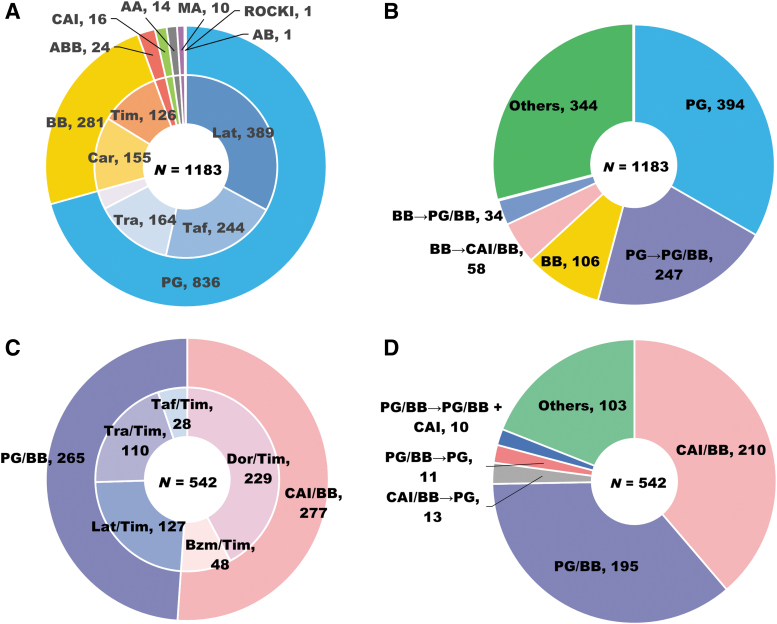
First-line drugs and treatment patterns over the 12-month postindex period. Drug class and generic names of first-line monotherapy **(A)** and treatment patterns **(B)** in the guideline-compliant cohort. Drug class and generic names of first-line fixed-combination therapy **(C)** and treatment patterns **(D)** in the guideline-noncompliant cohort. AA, α2 agonist; AB, α1 blocker; ABB, αβ blocker; BB, β blocker; Bzm, brinzolamide; CAI, carbonic anhydrase inhibitor; Car, carteolol; Dor, dorzolamide; Lat, latanoprost; MA, muscarinic agonist; PG, prostaglandin analog, ROCKI, rho-associated coiled kinase inhibitor; Taf, tafluprost; Tim, timolol; Tra, travoprost.

The eye drops prescribed at the index date are summarized in Fig. 2. According to drug class, PG and BB were commonly prescribed in the GL-compliant cohort for 836 (70.7%) and 281 (23.8%) patients, respectively, whereas other treatments (ABB, CAI, AA, MA, and ROCKI) were rarely used (Fig. 2A). The top 3 commonly used eye drops according to generic name were latanoprost [389 (32.9%)], tafluprost [244 (20.6%)], and travoprost [164 (13.9%)] (Fig. 2A). Common treatment patterns from the index date to 12 months are shown in Fig. 2B. According to drug class, typical switching patterns were from PG to PG/BB [247 (20.9%)], BB to CAI/BB [58 (4.9%)], and BB to PG/BB [34 (2.9%)]. In the GL-noncompliant cohort, CAI/BB [277 (51.1%)] and PG/BB [265 (48.9%)] were used by drug class, and the top 3 commonly used eye drops according to generic name were dorzolamide/timolol [229 (42.3%)], latanoprost/timolol [127 (23.4%)], and travoprost/timolol [110 (20.3%)] (Fig. 2C). The majority of patients received the same drug class over the 12-month postindex period. The proportion of the patients switching to other fixed-combination treatments was low (Fig. 2D).

Regarding the primary outcome of medication adherence, the mean PDC over the 12-month postindex period was 60.5% among all patients, 60.9% in the GL-compliant cohort, and 59.7% in the GL-noncompliant cohort (Table 2). High adherence (PDC ≥80%) was observed in 41.6% of all patients, 40.8% in the GL-compliant cohort, and 43.4% in the GL-noncompliant cohort. No significant difference was observed between the 2 cohorts. However, a comparison between the representative treatment patterns of the GL-compliant cohort and GL-noncompliant cohort (switching from latanoprost to latanoprost/timolol vs. latanoprost/timolol) revealed that the proportion of patients with PDC ≥80 was significantly higher in the latanoprost/timolol subset (*P* = 0.0061), although this did not reach statistical significance for the mean value (Table 3).

**Table 2.** Adherence to First-Line Therapy of the Guidelines-Compliant and -Noncompliant Cohorts During the 12-Month Postindex Period

	All patients* N = *1725	GL-compliant cohort* N = *1183	GL-noncompliant cohort* N = *542	
PDC, mean ± SD (%)	60.5 ± 34.6	60.9 ± 34.0	59.7 ± 35.8	P = 0.5131^*^
	*n* (%)	*n* (%)	*n* (%)	
80+% PDC	718 (41.6%)	483 (40.8%)	235 (43.4%)	*P* = 0.3225^#^
<80% PDC	1007 (58.4%)	700 (59.2%)	307 (56.6%)	
60–79% PDC	190 (11.0%)	143 (12.1%)	47 (8.7%)	
40–59% PDC	217 (12.6%)	170 (14.4%)	47 (8.7%)	
20–39% PDC	251 (14.6%)	177 (15.0%)	74 (13.7%)	
<20% PDC	349 (20.3%)	210 (17.8%)	139 (25.6%)	

^*^ *t*-test between GL-compliant and -noncompliant cohorts.

# *χ*^2^ test between GL-compliant and -noncompliant cohorts.

A *P* value <0.05/6 ( = 0.00833) was considered statistically significant (by Bonferroni correction).

PDC, proportion of days covered.

**Table 3.** Adherences to First-Line Latanoprost Therapy of the Subset of Switch from Latanoprost to Latanoprost/Timolol in the Guideline-Compliant Cohort and to First-Line Latanoprost/Timolol Therapy in the Noncompliant Cohorts During the 12-Month Postindex Period

	All patients* N = *316	Latanoprost* N = *189	Latanoprost/timolol* N = *127	
PDC, mean ± SD (%)	65.3 ± 33.5	62.9 ± 32.8	68.8 ± 34.3	P = 0.1287^*^
	*n* (%)	*n* (%)	*n* (%)	
80+% PDC	147 (46.5%)	76 (40.2%)	71 (55.9%)	*P* = 0.0061^#^
<80% PDC	169 (53.5%)	113 (59.8%)	56 (44.1%)	
60–79% PDC	36 (11.4%)	26 (13.8%)	10 (7.9%)	
40–59% PDC	44 (13.9%)	34(18.0%)	10 (7.9%)	
20–39% PDC	40 (12.7%)	24 (12.7%)	16 (12.6%)	
<20% PDC	49 (15.5%)	29 (15.3%)	20 (15.7%)	

^*^ *t*-test (Latanoprost vs. Latanoprost/timolol).

# *χ*^2^ test (Latanoprost vs. Latanoprost/timolol).

A *P* value <0.05/6 ( = 0.00833) was considered statistically significant (by Bonferroni correction).

**FIG. 3.**
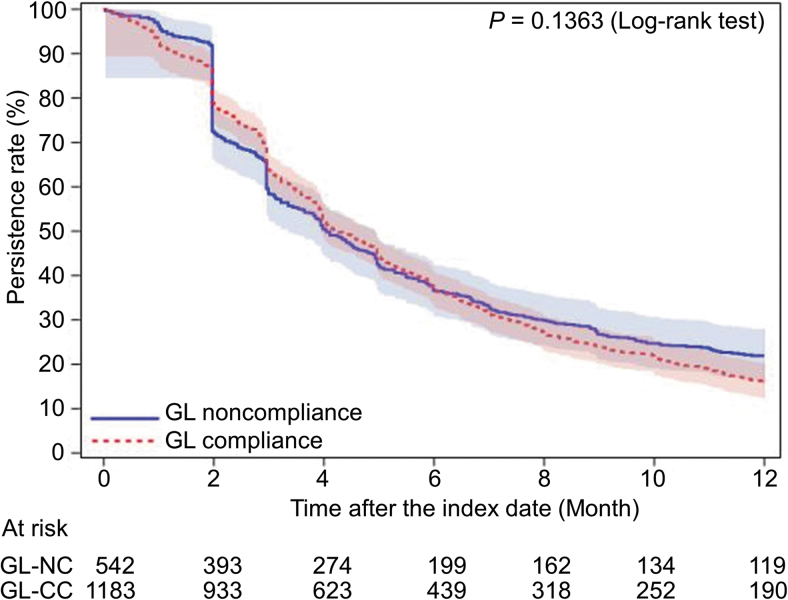
The Kaplan–Meier survival curves for the first-line treatment persistence in the guideline-compliant cohort compared with the guideline-noncompliant cohort. GL, guidelines; GL-CC, guideline-compliant cohort; GL-NC, guideline-noncompliant cohort.

**FIG. 4.**
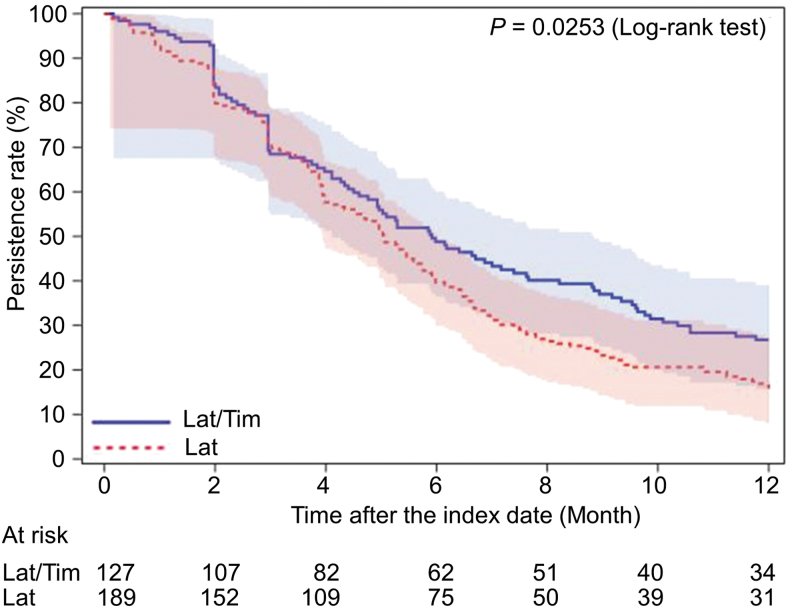
The Kaplan–Meier survival curves for first-line latanoprost therapy of the subset that switched from latanoprost to latanoprost/timolol in the guideline-compliant cohort and for first-line latanoprost/timolol therapy in the guideline-noncompliant cohort. Bars represent the Hall–Wellner bands. Lat, latanoprost; Tim, timolol.

As for the other primary outcome regarding persistence over 12 months, the Kaplan–Meier curves for the GL-compliant and noncompliant cohorts are presented in Fig. 3. No significant difference was noted between the cohorts (*P* = 0.1363). The persistence rates (95% CI) at 6 and 12 months postindex were 37.1% (34.4–39.9) and 16.0% (14.0–18.1), respectively, in the GL-compliant cohort, and 36.7% (32.7–40.8) and 22.0% (18.6–25.5%), respectively, in the GL-noncompliant cohort. Thus, the persistence rate over a longer period showed a better trend for the GL-noncompliant cohort. Compared with the subset of patients switching from latanoprost to latanoprost/timolol in the GL-compliant cohort, the latanoprost/timolol subset in the GL-noncompliant cohort showed a tendency toward higher persistence, but this did not reach statistical significance (Fig. 4).

The annual glaucoma consultation rates are summarized in Table 4. Patients in the GL-compliant and noncompliant cohorts underwent general eye examinations 5.23 and 4.76 times on average for 12 months. In contrast, most patients in the GL-compliant (97.4%) and -noncompliant (97.8%) cohorts did not take a visual field test, and around half of the patients underwent optical coherence tomography once. There was no tendency for the GL-compliant cohort to have more visits or better PDCs.

**Table 4.** Annual Consultation Rate After the Index Date

	Ophthalmoscopy and tonometry		Perimetry		Optical coherence tomography	
	GL-CC (*N = *1183)	GL-NC (*N = *542)	GL-CC (*N = *1183)	GL-NC (*N = *542)	GL-CC (*N = *1183)	GL-NC (*N = *542)
Mean (SD)	5.23 ± 3.21	4.76 ± 3.19	0.05 ± 0.37	0.03 ± 0.25	1.11 ± 1.67	1.26 ± 1.80
None, *n* (%)	83 (7.0%)	52 (9.6%)	1152 (97.4%)	530 (97.8%)	539 (45.6%)	231 (42.6%)
1–3 times, *n* (%)	289 (24.4%)	156 (28.8%)	27 (2.3%)	12 (2.2%)	577 (48.8%)	267 (49.3%)
4–6 times, *n* (%)	437 (36.9%)	180 (33.2%)	3 (0.3%)	0	45 (3.8%)	25 (4.6%)
7–9 times, *n* (%)	225 (19.0%)	105 (19.4%)	1 (0.1%)	0	11 (0.9%)	16 (3.0%)
10–12 times, *n* (%)	149 (12.6%)	49 (9.0%)	0	0	11 (0.9%)	3 (0.6%)
>12 times, *n* (%)	0	0	0	0	0	0

Ophthalmoscopy: test of the retina, optic disk, and vitreous humor. Tonometry: intraocular pressure test. Perimetry: visual field test.

GL-CC, guidelines-compliant cohort; GL-NC, guidelines-noncompliant cohort.

A *P-*value <0.05/6 ( = 0.00833) was considered statistically significant (by Bonferroni correction).

Glaucoma surgery was performed within 12 months after the index date for the first time in 15 patients (1.3%) in the GL-compliant cohort and 10 patients (1.8%) in the GL-noncompliant cohort, and the number of surgery cases was 0.42 and 0.71/100 person-years of observation (PYO), respectively. There were no marked differences in characteristics between 2 cohorts (Supplementary Table S1).

Univariate analyses between the 2 cohorts according to each baseline characteristic identified factors for GL noncompliance, including γ-glutamyl transpeptidase (γ-GTP) (*P* = 0.0205), and asthma or chronic obstructive pulmonary disease (*P* = 0.0921) (Table 5). Multivariate analyses using the factors obtained from univariate analyses followed by variable selection in a stepwise manner identified only γ-GTP [odds ratio: 1.003 per one unit increase (95% CI: 1.000–1.006), *P* = 0.0205] as a factor for GL noncompliance (Table 5). However, 95% CI of odds ratio included 1 and γ-GTP was measured in only ∼60% of patients as shown in Table 1.

**Table 5.** Analysis on Factors and the Guideline Compliance or Noncompliance for All Subjects

Factors	Univariate	Multivariate	Odds ratio (95% CI)
	P-value	P-value	
Health care checkup values			
Sex	0.2253		
Age	0.6073		
Body weight	0.4984		
Height	0.9605		
Body mass index	0.4070		
Smoking	0.7936		
Medication for other indication	0.9851		
Fasting blood glucose	0.3827		
HbA1c	0.9152		
Urine glucose	0.8863		
Urine protein	0.1837		
Antihypertensive drug	0.4960		
Systolic blood pressure	0.9555		
Diastolic blood pressure	0.7423		
Total cholesterol	0.4904		
Triglycerides	0.6899		
HDL-C	0.5517		
LDL-C	0.2584		
Aspartate aminotransferase	0.7310		
Alanine aminotransferase	0.2273		
*γ*-GTP	0.0205	0.0205	1.003 (1.000–1.006)
Locations			
47 prefectures	0.9060		
8 regions	0.7315		
East and West Japan	0.8456		
City size	0.3207		
*Common comorbidities*			
Hypertension	0.7231		
Coronary artery disease	0.2804		
Angina pectoris	0.4002		
Acute myocardial infarction	0.2223		
Ischemic heart disease	0.1168		
Cardiac arrhythmia	0.7041		
Heart failure	0.5361		
Atherosclerosis or PAOD	0.6347		
Cerebral ischemia/chronic apoplexy	0.8719		
Cerebral infarction	0.5411		
Hyperlipidemia	0.6874		
Diabetes mellitus	0.5465		
Hypothyroidism	0.6608		
Water-electrolyte disorder	0.2470		
Mental disease or neurosis	0.4369		
Depression	0.8556		
Insomnia	0.4562		
Parkinson's disease	0.1108		
Gastrointestinal ulcer	0.9867		
Nephropathy or chronic renopathy	0.2905		
Kidney failure	0.2606		
Hepatopathy	0.6688		
Liver failure	0.5808		
Pneumonia	0.1431		
Asthma or COPD	0.0921		
Cancer	0.6707		
Anemia	0.1369		

CI, confidence interval.

## Discussion

To date, there have been no studies on GL compliance of physicians in new patients with glaucoma in Japan or other countries. This study revealed that most Japanese patients with glaucoma were treated in accordance with the GLs, but ∼5% of patients were prescribed a fixed-combination eye drop as first-line therapy. Although adherence to the therapy was moderate, there were no significant differences between the GL-compliant cohort and the GL-noncompliant cohort regarding the adherence and persistence to first-line therapy, annual glaucoma consultation, or annual glaucoma surgery. Therefore, first-line fixed-combination eye drops may be considered to be a convenient treatment option for glaucoma patients according to the patients' medical needs, especially for those who are already at the severe glaucoma stage at the initial glaucoma consultation and need early IOP lowering treatment, those who are relatively young and for whom it is important to delay blindness, or those who need personalized medication due to their lifestyle.

According to the recommendation found in GLs as first-line treatment, PGs, such as latanoprost, tafluprost, or travoprost, were commonly prescribed. The most common treatment pattern in the GL-compliant cohort over the 12-month postindex period was PG (33.1%) followed by PG switching to PG/BB (20.9%). However, few patients switched to other single drugs or add-on therapies. On the contrary, >70% of the GL-noncompliant cohort received CAI/BB (38.7%) and PG/BB (36.0%) over the 12-month postindex period.

The 12-month adherence to the first treatment was moderate in both cohorts and was similar between the 2 cohorts; the mean PDC was 60.9% in the GL-compliant cohort and 59.7% in the GL-noncompliant cohort at 12 months. Persistence over the 12-month postindex period did not differ, and the persistence rate of the first-line treatment was 16.0% in the GL-compliant cohort and 22.0% in the GL-noncompliant cohort. The persistence rates observed in this study were lower than those reported by a previous study (60.9%) in newly diagnosed Japanese patients.^17^ One possible explanation for these differences in the persistence rates is the differences in the definition of “discontinued.” In the previous study, the change of the initially prescribed glaucoma eye drops was not defined as “discontinued,”^17^ but it was defined so in this study. In the GL-compliant cohort, patients who switched to a fixed-combination therapy from a monotherapy as first-line therapy were considered to have discontinued their treatment. In the GL-noncompliant cohort, patients who switched to an unfixed-combination therapy or monotherapy from a fixed-combination therapy as first-line therapy were considered to have discontinued their treatment. Differences in the first dosing regimen (eg, single, unfixed combination, fixed combination) may be another reason for the differences among the studies' results. In the previous study, 94.1% of new patients with glaucoma were medicated with single glaucoma eye drops^17^; however, all the patients were medicated with a single (the GL-compliant cohort) or fixed combination (the GL-noncompliant cohort) in this study. Therefore, both studies evaluated different things; the previous study evaluated the persistence rate to total glaucoma medication use, whereas this study evaluated the persistence rate to the first-line glaucoma medication use. A systematic review summarized the results from 14 published studies focusing on the persistence with glaucoma medication use, and reported that only 31% of new patients with glaucoma remained persistent 12 months after starting therapy.^18^ Taken together, it is likely that patients in this study included those requiring a change in treatment plan due to relatively severe glaucoma stage at the index date or those who required early IOP lowering treatment.

Patients in both cohorts frequently received glaucoma consultations; the mean number of annual consultations with ophthalmoscopy and tonometry was 5.23 times in the GL-compliant cohort and 4.76 times in the GL-noncompliant cohort. A similar tendency was observed for other examinations.

Glaucoma surgery was observed to be performed at a certain rate in the GL-noncompliant cohort (0.71/100 PYO) and the GL-compliant cohort (0.42/100 PYO) for the 12-month postindex period. These surgery rates are likely due to the fact that patients who required early treatment but also required consideration of ocular allergy to eye drops, who had difficulty visiting the hospital, and who had cataract surgery were started with a fixed-combination drug and then immediately underwent surgery when they experienced difficulty in achieving reduction in IOP to prevent damage to the optic nerve.

No key factor was identified as being associated with GL noncompliance. This suggests that patients may be treated with either first-line monotherapy or fixed-combination therapy. Furthermore, physicians can consider prescribing fixed-combination agents according to the patient's convenience depending on their characteristics, such as lifestyle and medication adherence. However, this study did not consider severity of glaucoma as these data were not available in the database. Although we compared data between newly diagnosed patients who were prescribed first-line monotherapy and those prescribed a fixed-combination therapy, it remains unclear whether there was a comparable distribution of severity of glaucoma among the 2 cohorts. Physicians may decide on a prescription according to the severity of the disease; however, a fixed-combination therapy would be appropriate since dual therapy is more effective than monotherapy.^9,19^

In conclusion, the patients' adherence and persistence to first-line therapy were not very high, but no significant differences were found between the GL-noncompliant cohort and the GL-compliant cohort. About 5% among all the physicians prescribed a fixed dose combination as first-line therapy, which did not comply with the GL. Furthermore, the annual incidence of glaucoma consultation or glaucoma surgery was similar. No factors associated with GL noncompliance were noted among newly diagnosed Japanese patients with glaucoma. Thus, fixed-combination eye drops could be used as first-line therapy as well as second-line therapy for glaucoma. A first-line fixed-combination option would be useful for personalized medication in view of severity of glaucoma in addition to improvement of adherence and health care by understanding patient preferences in daily practice.

## Supplementary Material

Supplemental data
