# Supplementary material for: Adherence and Persistence with First-Line Therapy and Compliance with Glaucoma Guidelines Using Japanese Health Care/Pharmacy Claims Database
Source: J Ocul Pharmacol Ther. 2021 Jan 11;37(1):35–44. doi: 10.1089/jop.2020.0096 (PMC8045955; doi:10.1089/jop.2020.0096)
Supplement: Supplemental data [file Supp_TableS1.docx]

Supplemental Table 1. Baseline and clinical characteristics of patients undergoing glaucoma surgery after the index date

| *Variable* | *GL-compliant cohort (N = 15)* | |  | *GL-noncompliant cohort (N = 10)* | |
| --- | --- | --- | --- | --- | --- |
|  | *n* | *Percentage or*  *mean ± SD* |  | *n* | *Percentage or*  *mean ± SD* |
| Sex, male (%) | 8 | 53.3 |  | 6 | 60.0 |
| Age (years) | 15 | 53.9 ± 13.3 |  | 10 | 54.7 ± 12.4 |
| Body weight (kg) | 7 | 54.1 ± 8.5 |  | 5 | 62.0 ± 7.2 |
| Height (cm) | 7 | 162.5 ± 11.8 |  | 5 | 165.6 ± 6.2 |
| Body mass index (kg/m^2^) | 7 | 20.5 ± 2.8 |  | 5 | 22.8 ± 3.7 |
| Smoking (%) |  |  |  |  |  |
| Yes | 1 | 6.7 |  | 1 | 10,0 |
| No | 6 | 40.0 |  | 4 | 40.0 |
| Medication for other indications (%) |  |  |  |  |  |
| Yes | 2 | 13.3 |  | 1 | 10.0 |
| No | 5 | 33.3 |  | 4 | 40.0 |
| Fasting blood glucose (mg/dL) | 7 | 100.7 ± 23.0 |  | 5 | 92.4 ± 12.3 |
| HbA1c (NGSP) | 6 | 5.7 ± 0.3 |  | 5 | 5.4 ± 0.3 |
| Urine glucose (%) |  |  |  |  |  |
| Negative (−) | 7 | 46.7 |  | 5 | 50.0 |
| Positive (≥±) | 0 | 0 |  | 0 | 0 |
| Urine protein (%) |  |  |  |  |  |
| Negative (−) | 7 | 46.7 |  | 5 | 50.0 |
| Positive (≥±) | 0 | 0 |  | 0 | 0 |
| Antihypertensive drug (%) |  |  |  |  |  |
| Yes | 1 | 6.7 |  | 1 | 10.0 |
| No | 6 | 40.0 |  | 4 | 40.0 |
| Systolic blood pressure (mm Hg) | 7 | 115.7 ± 12.3 |  | 5 | 128.2 ± 18.7 |
| Diastolic blood pressure (mm Hg) | 7 | 74.4 ± 9.0 |  | 5 | 78.8± 9.8 |
| Total cholesterol (mg/dL) | 4 | 215.3 ± 63.3 |  | 1 | 151.0 ± n/a |
| Triglycerides (mg/dL) | 7 | 77.4 ± 23.5 |  | 5 | 103.6 ± 58.1 |
| HDL-C (mg/dL) | 7 | 68.1 ± 25.6 |  | 5 | 58.2 ± 19.1 |
| LDL-C (mg/dL) | 7 | 120.6 ± 31.3 |  | 5 | 120.0± 24.7 |
| Aspartate aminotransferase (U/L) | 7 | 20.3 ± 3.7 |  | 5 | 20.2 ± 4.6 |
| Alanine aminotransferase (U/L) | 7 | 15.7 ± 5.0 |  | 5 | 21.0 ± 7.8 |
| γ-GTP (U/L) | 7 | 18.7 ± 7.1 |  | 5 | 32.0 ± 17.2 |
| Common comorbidities (%) |  |  |  |  |  |
| Hypertension | 6 | 40.0 |  | 1 | 10.0 |
| Coronary artery disease | 0 | 0 |  | 1 | 10.0 |
| Angina pectoris | 0 | 0 |  | 1 | 10.0 |
| Acute myocardial infarction | 0 | 0 |  | 0 | 0 |
| Ischemic heart disease | 0 | 0 |  | 0 | 0 |
| Cardiac arrhythmia | 1 | 6.7 |  | 2 | 20.0 |
| Heart failure | 1 | 6.7 |  | 0 | 0 |
| Atherosclerosis or PAOD | 0 | 0 |  | 1 | 10.0 |
| Cerebral ischemia/chronic apoplexy | 1 | 6.7 |  | 1 | 10.0 |
| Cerebral infarction | 0 | 0 |  | 0 | 0 |
| Varicose veins of lower limb | 0 | 0 |  | 0 | 0 |
| Hyperlipidemia | 5 | 33.3 |  | 2 | 20.0 |
| Diabetes mellitus | 7 | 46.7 |  | 3 | 30.0 |
| Hypothyroidism | 1 | 6.7 |  | 2 | 20.0 |
| Water-electrolyte disorder | 1 | 6.7 |  | 0 | 0 |
| Mental disease or neurosis | 0 | 0 |  | 1 | 10.0 |
| Depression | 0 | 0 |  | 1 | 10.0 |
| Insomnia | 0 | 0 |  | 1 | 10.0 |
| Parkinson disease | 0 | 0 |  | 0 | 0 |
| Dementia | 0 | 0 |  | 0 | 0 |
| Gastrointestinal ulcer | 1 | 6.7 |  | 0 | 0 |
| Nephropathy or chronic renopathy | 0 | 0 |  | 0 | 0 |
| Kidney failure | 0 | 0 |  | 1 | 10.0 |
| Incontinentia | 0 | 0 |  | 0 | 0 |
| Hepatopathy | 1 | 6.7 |  | 1 | 10.0 |
| Liver failure | 0 | 0 |  | 0 | 0 |
| Pneumonia | 0 | 0 |  | 1 | 10.0 |
| Asthma or COPD | 1 | 6.7 |  | 1 | 10.0 |
| Cancer | 4 | 26.7 |  | 4 | 40.0 |
| Anemia | 3 | 20.0 |  | 1 | 10.0 |
| Locations |  |  |  |  |  |
| 8 regions (%) |  |  |  |  |  |
| Hokkaido | 0 | 0 |  | 1 | 10.0 |
| Tohoku  Kanto | 0 | 0 |  | 0 | 0 |
| Kanto | 9 | 60.0 |  | 5 | 50.0 |
| Chubu | 1 | 6.7 |  | 1 | 10.0 |
| Kinki | 2 | 13.3 |  | 2 | 20.0 |
| Chugoku | 1 | 6.7 |  | 0 | 0 |
| Shikoku | 0 | 0 |  | 0 | 0 |
| Kyushu | 2 | 13.3 |  | 1 | 10.0 |
| Areas (%) |  |  |  |  |  |
| East Japan | 9 | 60.0 |  | 6 | 60.0 |
| West Japan | 6 | 40.0 |  | 4 | 40.0 |
| City size (%) |  |  |  |  |  |
| Tokyo and all GODMCs | 11 | 73.3 |  | 10 | 100.0 |
| Other than GODMCs | 4 | 26.7 |  | 0 | 0 |

GL, guidelines; NGSP, National Glycohemoglobin Standardization Program; HDL-C, high-density lipoprotein cholesterol; LDL-C, low-density lipoprotein cholesterol; γ-GTP, γ-glutamyl transpeptidase; PAOD, peripheral arterial occlusive disease; COPD, chronic obstructive pulmonary disease; GODMCs, government ordinance-designed major cities.
